# Surgical Drainage and Simultaneous Sinus Floor Augmentation in Patients with Chronic Maxillary Sinusitis

**DOI:** 10.3390/medicina60081256

**Published:** 2024-08-02

**Authors:** Won-Bae Park, Hye-Kyoung Seol, Seungil Shin, Ji-Youn Hong

**Affiliations:** 1 Private Practice in Periodontics and Implant Dentistry, Seoul 02771, Republic of Korea; wbpdds@naver.com; 2 Department of Dentistry, Graduate School, Kyung Hee University, Seoul 02447, Republic of Korea; hkseol126@naver.com; 3 Department of Periodontology, College of Dentistry, Kyung Hee University Dental Hospital, Kyung Hee University, Seoul 02447, Republic of Korea; shin.dmd@khu.ac.kr

**Keywords:** dental implant, drainage, maxillary sinus floor augmentation, maxillary sinusitis

## Abstract

Chronic maxillary sinusitis accompanied by severe thickening of the sinus mucosa, blockage of the ostium, and patient-reported symptoms requires preoperative assessment and treatment by an otolaryngologist before maxillary sinus floor augmentation (MSFA). Prescription of antibiotics and nasal saline irrigation are the first choice of treatment; however, endoscopic sinus surgery is considered when the treatment’s effect is limited and drug resistance is observed. Nevertheless, MSFA performed in the presence of sinus pathologies have been reported to have favorable results when the lesions are managed properly. This report presents cases of two patients who required MSFA but were diagnosed with chronic maxillary sinusitis (case 1 with nasal sinusitis and case 2 with dental sinusitis). After 2 weeks of antibiotic therapy, endoscopic surgery was recommended due to minimal changes in the size of the sinus lesion; however, the patients refused because of improved self-reported symptoms. Therefore, intraoral surgical drainage was planned as an alternative treatment. A large bony window was prepared at the lateral wall of the maxillary sinus, and a long intentional incision was made to improve access for the suction tip in various directions and depths into the sinus cavity. Thorough suction of the purulent exudate and saline irrigation were performed through this access. The size of the perforated area was reduced along with the elevation of the Schneiderian membrane from the sinus floor, and simultaneous bone grafting with implant placement was performed. Prosthesis was delivered after 6–8 months. At 1-year follow-up after loading, favorable outcomes of implant survival and maintenance of augmented bone height were observed, with no recurrence of postoperative sinusitis. Within the limitations of the present case report, thorough sinus drainage and saline irrigation during maxillary sinus floor augmentation resolved sinus infection in patients with chronic maxillary sinusitis with short-term clinical outcomes.

## 1. Introduction

Ridge atrophy with a pneumatized sinus, resulting in a lack of available bone height, is a great challenge for clinicians when rehabilitating the edentulous posterior area with dental implants in the maxilla. Maxillary sinus floor augmentation (MSFA) using the lateral window approach, first introduced by Boyne and James [1], has been successfully used to treat severely pneumatized maxillary sinuses, and long-term clinical studies have shown predictable results in implant survival [2,3]. Despite the reliability and safety of MSFA, the lateral window approach is associated with several intraoperative and postoperative complications, including perforation of the Schneiderian membrane, acute or chronic paranasal sinusitis, infections, and graft failure [4,5]. The incidence of complications is low and can be solved readily, but MSFAs can occasionally become serious [5]. Furthermore, preoperative sinusitis significantly influences the development of postoperative sinusitis [6]. Therefore, assessment of the presence of preexisting sinus pathologies and anatomic variations is crucial, and an otorhinolaryngology consultation should be considered when preliminary control of sinus infection and correction of pathologic findings are necessary.

During preoperative assessment in dental clinics, a detailed history should be obtained from the patient, including any previous nasal trauma or surgery, nasal obstruction, allergic symptoms, smell and taste dysfunction, and sinus infections [7]. Radiological assessment using computed tomography (CT) or wide-range cone-beam CT is also necessary to visualize soft-tissue lesions, to assess the status of the inner wall and osteomeatal complex, and to evaluate the height and volume of the maxillary sinus [8]. Maxillary sinus pathologies that can be identified on CT images include mucosal thickening, cysts or solitary polyps, acute or chronic rhinosinusitis, and paranasal malignancy. The health status of the maxillary sinus can be indicated by sinus mucosa thickness, mucociliary clearance, ostium patency, infundibular passage, and patient-reported symptoms, which play important roles in maintaining the physiology and homeostasis of the maxillary sinus [9,10].

Chronic maxillary sinusitis (CMS) is associated with rhinosinusitis of nasal origin and odontogenic maxillary sinusitis of dental origin. Symptoms of rhinosinusitis, including nasal blockage, obstruction, congestion, and nasal discharge, persist for more than 12 weeks, and unilateral sinus involvement suggests fungal or odontogenic sinusitis [11,12]. Fungal rhinosinusitis typically shows distinctive features of mottled calcified foci [8]. Odontogenic maxillary sinusitis occurs secondary to adjacent infectious dental lesions such as endodontic or periodontal disease, oroantral fistula, impaction of foreign bodies related to dental treatment, or iatrogenic injury from dental procedures [12]. Its incidence is reported in a range from 10% to 40% of all maxillary sinusitis cases [7,12,13,14]. Chronic rhinosinusitis (CRS) is treated with antibiotics including penicillins, cephalosporins, fluoroquinolones, or aminoglycosides [15], and nasal saline irrigation is used to aid mucous membrane regeneration [16]. Complete resolution may take 3–6 months; however, functional endoscopic sinus surgery (FESS) is recommended when medical treatment shows limited improvements due to underlying anatomical conditions or drug resistance [8,17,18].

Some clinical studies have introduced surgical interventions that combine MSFA with FESS to treat coexisting sinus pathologies [19,20,21]. This approach is advantageous for direct access and visualization of the lesion through a small opening at the lateral wall of the sinus; however, it requires special instruments and settings with interdisciplinary team approaches. Patients generally require hospitalization and general anesthesia and hesitate to undergo surgery due to economic burdens. Other approaches involve performing intraoral sinus drainage during the lateral window approach with simultaneous MSFA, which have shown reliable clinical outcomes in terms of considerable reduction in soft tissue lesions and sinus opacification, even in cases with severe preoperative mucosal thickening or ostium blockage [9,22,23]. However, the clinical evidence for intraoral drainage with MSFA in CMS is limited. The present case report introduces intraoral sinus drainage simultaneously with MSFA in patients with CMS with opacified lesions in the entire unilateral maxillary sinus and describes the clinical outcomes during a short-term observation period.

## 2. Case Presentation

This report presents the cases of two patients who sought dental implant treatment at a private clinic specialized in periodontics and implant dentistry. Both patients presented with complete opacification of the entire cavity of the unilateral maxillary sinus on preoperative radiographs. The patients were referred to an otolaryngologist for the assessment of sinus pathologies, and both were diagnosed with CMS. Antibiotics including amoxicillin/potassium clavulanate (Augmentin 625 mg, Ilsung Pharmaceuticals Co., Ltd., Seoul, Republic of Korea) were prescribed for 2 weeks; however, the extent of the opacified lesion showed only minimal changes on cone beam computed tomography (CBCT) after antibiotic treatment. Although FESS was recommended to remove the lesion, both patients declined additional surgery as the symptoms of CMS improved. Therefore, we planned to perform sinus drainage through a lateral window opening for the MSFA. Both patients were informed of the risks including postoperative graft infection, sinusitis and graft failure as well as the benefits regarding shorter treatment period, lower cost, and avoidance of hospitalization with additional surgery. Written informed consent was received from the patients.

### 2.1. Case 1

A 59-year-old male smoker patient who had been wearing a complete denture on the maxilla and a removable partial denture on the mandible for 15 years visited the clinic with a desire to have implant-supported protheses. During history-taking, the patient reported that he had mild chronic nasal congestion and had been receiving treatment for rhinitis for approximately 4 months before visiting the dental clinic. There were no other systemic diseases reported from the patient. After consultation with an otolaryngologist, the patient was diagnosed with CMS and was prescribed antibiotics for 2 weeks. The size of the opacified lesion in the right maxillary sinus rarely changed, and FESS was recommended to resolve the lesion. However, the patient refused to undergo surgery as the symptoms related to CMS improved, and he was sent back to the dental clinic. Intraoral intervention to remove the sinus pathology during lateral sinus floor elevation was decided as an alternative treatment which was performed by an oral surgeon (W.-B.P.).

**Figure 1:**
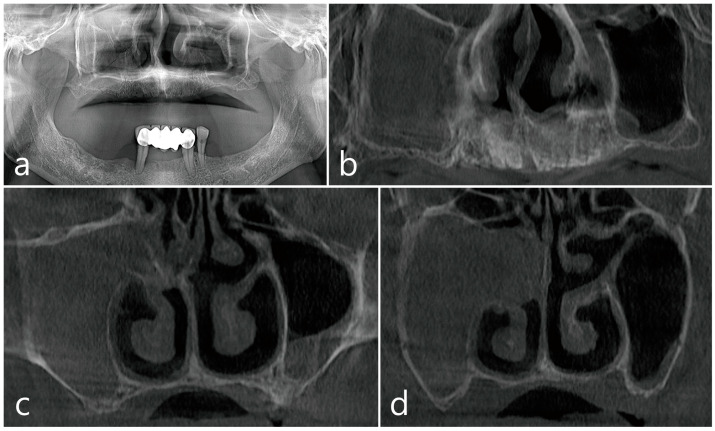
Case 1. Preoperative radiographs. (**a**) Severe atrophy of the residual alveolar bone shown in the panoramic image. (**b**) Panoramic image of CBCT scan showing complete opacification in the unilateral right maxillary sinus. (**c**,**d**) Coronal images of CBCT showing the blockage of natural ostium and severely thickened mucosa occupying up to the middle meatus. CBCT; cone beam computed tomography.

MSFAs were required in the maxillary sinus because of severe atrophy of the residual ridge accompanied by pneumatized sinuses (Figure 1a). A panoramic CBCT image (Rainbow^TM^, Dentium, Suwon, Republic of Korea) obtained before surgery showed unilateral sinusitis with complete opacification of the right maxillary sinus (Figure 1b). On the coronal CBCT image, the natural ostium was blocked, and the nasal polyp occupied the middle meatus (Figure 1c,d).

**Figure 2:**
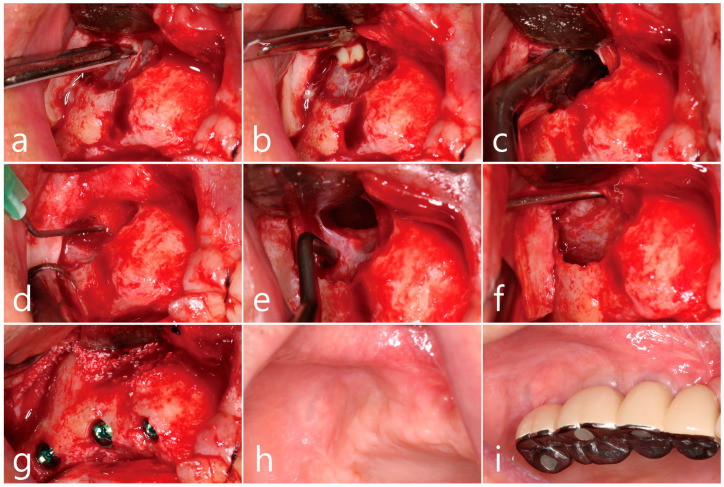
Case 1. Surgical procedures. (**a**) Mucoperiosteal flap reflection and preparation of lateral window sized approximately 1.0 cm × 1.5 cm in right posterior maxilla. (**b**) Intentional horizontal incision in the upper third of the Schneiderian membrane for sinus drainage and pus discharge through the perforated site. (**c**) Insertion of the suction tip into the maxillary sinus to remove inflammatory exudate and pus. (**d**) Sufficient saline irrigation until the inflammatory exudate stopped flowing out. (**e**) Careful separation of the Schneiderian membrane from the sinus floor. (**f**) Collapse of perforated site with reduction in size along with the elevation of Schneiderian membrane. (**g**) Sinus floor augmentation with bone graft materials and placement of implants. (**h**) A well-healed surgical site observed 6 months after surgery. (**i**) One-year follow-up after prosthetic loading.

Under local anesthesia, a full thickness mucoperiosteal flap with vertical releasing incisions was reflected in the maxillary posterior region, and a bony window was prepared using a surgical round bur. The lateral bony window of approximately 1.5 cm × 2.0 cm in size was carefully prepared without damaging the Schneiderian membrane. A long horizontal incision was intentionally made for sinus drainage using a #15 Bard–Parker blade in the upper third of the exposed Schneiderian membrane (Figure 2a). Purulent exudate was discharged through the incised perforation (Figure 2b). A suction tip was inserted into the maxillary sinus cavity through the perforation, and the inflammatory exudate and pus were drained. The suction tip approached sufficiently far in every direction for thorough drainage (Figure 2c). Sufficient saline irrigation was performed until the inflammatory exudates and residues were no longer identified (Figure 2d). After irrigation, the Schneiderian membrane was carefully separated and elevated from the sinus floor using sinus elevators (Genoss, Suwon, Republic of Korea) (Figure 2e). The perforated area was reduced in size and relocated further buccally during the elevation of the Schneiderian membrane, as the tension on the mucous membrane decreased and mucosal folding became apparent (Figure 2f). No additional repairs were performed at the perforation site.

The sinus floor was augmented with xenogenic bone graft material (Osteon^TM^ Xeno, Genoss, Suwon, Republic of Korea) with the aid of a Prichard elevator to prevent displacement of the graft particles into the sinus cavity (Figure 2g). Three SLA (sandblasted, large grid, acid-etched)-textured implants (Ø 3.8 × 10 mm, Implantium, Dentium, Suwon, Republic of Korea) were simultaneously placed, and the flap was primarily closed without additional membrane coverage at the lateral window opening. For postoperative care, systemic antibiotics (ciprofloxacin [500 mg]; Ildong Pharmaceutical Co., Seoul, Republic of Korea) and nonsteroidal antiinflammatory drugs (Anaprox (275 mg); Chong Kun Dang Pharmaceutical Co., Seoul, Republic of Korea) were prescribed for 2 weeks. The patient was advised to refrain from blowing his nose postoperatively. Postoperative healing was uneventful, except for transient nasal bleeding, facial swelling, and hematoma. A well-healed surgical site was observed 6 months after the surgery (Figure 2h), and closure of the lateral window with regenerated hard tissue was observed during uncovering of the implant. The prosthesis was delivered 2 months after implant uncovering and was well maintained, with no clinical issues noted after 1 year of loading (Figure 2i).

**Figure 3:**
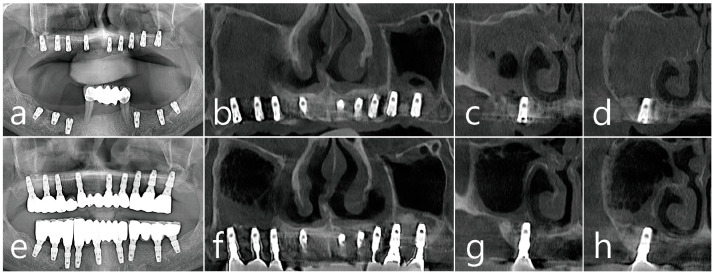
Case 1. Postoperative radiographs. (**a**) Panoramic radiograph of the right maxillary posterior area after surgical drainage, sinus floor augmentation, and implant placement. Panoramic (**b**) and coronal (**c**,**d**) images of CBCT obtained immediately after the surgery. Nasal polyps were noted. (**e**) Panoramic radiograph after 1-year of loading. No crestal bone loss was observed. Panoramic (**f**) and coronal (**g**,**h**) images of CBCT after 1-year of loading. Limited mucosal thickening lining the augmented sinus floor was shown. Patency of the natural ostium without nasal polyp was shown. CBCT; cone beam computed tomography.

Panoramic radiographs were obtained after surgical drainage, sinus floor augmentation, and implant placement in the right posterior maxilla (Figure 3a,b). A nasal polyp was observed in the coronal CBCT images obtained immediately after the surgery (Figure 3c,d). No specific displacement of the bone-graft materials into the sinus cavity was observed over the augmented bone surrounding the apex of the implant fixtures. In the panoramic radiograph obtained 1 year after prosthetic loading, maintenance of the augmented sinus floor and absence of specific peri-implant crestal bone loss was observed (Figure 3e). In panoramic and coronal CBCT images, sinus opacification, which previously occupied the whole cavity in the right maxilla, was resolved, and a slightly limited membrane thickening was noted on the augmented floor (Figure 3f–h).

### 2.2. Case 2

**Figure 4:**
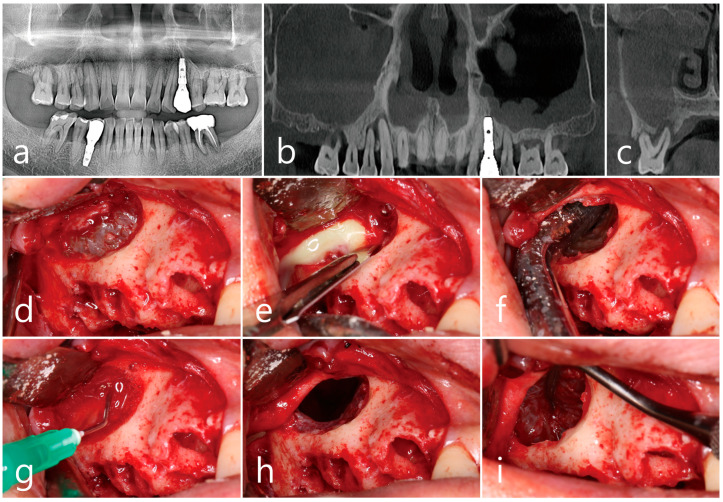
Case 2. (**a**) Preoperative panoramic radiograph showing the apical lesion at right maxillary canine, first premolar, and molar teeth. Panoramic (**b**) and coronal (**c**) images of CBCT showing the apical lesion around palatal root of the right maxillary first molar and opacified sinus cavity. Surgical procedures including (**d**) extraction of teeth, flap reflection, and lateral window opening; (**e**) intentional incision in the upper part of the exposed Schneiderian membrane and pus discharge; (**f**) sufficient drainage of purulent exudate using suction tip; (**g**) thorough saline irrigation; (**h**) a well-cleaned sinus cavity observed; (**i**) careful detachment of the Schneiderian membrane from the sinus floor and elevation. CBCT; cone beam computed tomography.

A 55-year-old male smoker patient with the chief complaints of severe tooth mobility and pain on chewing at the right maxillary posterior region visited the dental clinic for the treatment. In the history-taking, the patient had hypertension which was controlled well by medication, and he had been experiencing nasal discharge, halitosis, and bad odor from the nose when breathing for several months. The patient was prescribed medication and nasal irrigation when the symptoms increased. Preoperative panoramic radiography revealed loss of periodontal support and bone level reaching the root apex of the maxillary right canine and first premolar teeth. The right maxillary first molar showed furcation involvement and severe exposure of the palatal root, with vertical tooth mobility (Figure 4a). In the panoramic and coronal CBCT images, opacification of the entire sinus cavity in the right maxilla was observed, with an apically involved lesion of the maxillary right first molar adjacent to the sinus floor, which was presumed to be the source of infection for sinus pathology (Figure 4b,c). The patient was referred to an otolaryngologist, and the sinus lesion was diagnosed as CMS of dental origin. Antibiotics were prescribed for 2 weeks, and FESS was recommended. However, the patient refused to undergo additional medical surgery, and it was decided to treat the maxillary sinus lesion using the lateral window approach, as described in Case 1. Four teeth, including the maxillary right canine, first and second premolars, and first molar, were extracted, and the mucoperiosteal flap with vertical releasing incisions was reflected. A lateral bony window sized approximately 1.5 cm × 2.0 cm was prepared to access the Schneiderian membrane (Figure 4d). An intentional membrane perforation was made (Figure 4e), and the inflammatory exudate was drained followed by thorough irrigation (Figure 4f,g). Membrane detachment and elevation were carefully performed at the sinus floor (Figure 4h,i).

**Figure 5:**
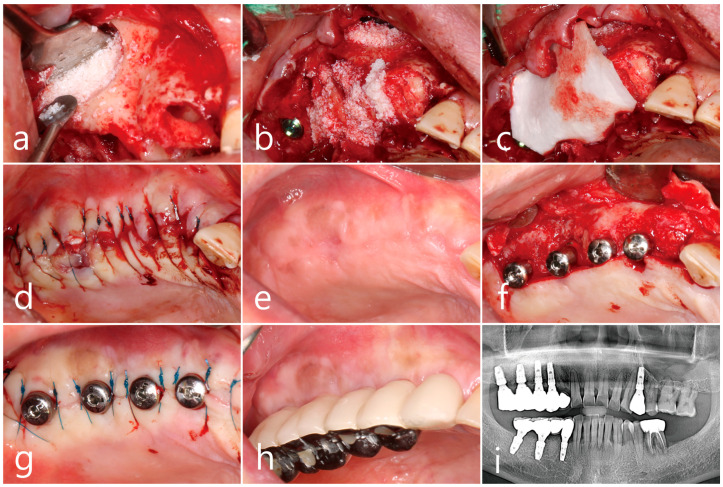
Case 2. Surgical procedures including (**a**) sinus floor augmentation with xenograft with insertion of a Prichard elevator to protect perforated site, (**b**) implant placement and filling of small peri-implant gap defects with graft materials, (**c**) implants covered with a resorbable collagen membrane, (**d**) flap closure. (**e**) Clinical findings 6 months after surgery. (**f**) Uncovering 6 months after the surgery and additional implant placement at right maxillary second premolar. (**g**) Flap closure around healing abutments. (**h**) Prosthesis delivered after 2 months. (**i**) Panoramic radiograph after 1 year of loading. No change in crestal bone level around the implant was observed. CBCT; cone beam computed tomography.

A xenograft (Osteon^TM^ Xeno; Genoss, Suwon, Republic of Korea) was placed on the floor of the sinus cavity (Figure 5a), and three SLA-textured implants (Implantium, Dentium, Suwon, Republic of Korea) were placed at the maxillary right first premolar site (Ø 3.8 × 12 mm) and first and second molar sites (Ø 4.3 × 10 mm and Ø 4.8 × 10 mm, respectively). The implants were placed 1.0–2.0 mm subcrestal and peri-implant gap defects were filled with xenograft (Osteon^TM^ Xeno, Genoss, Suwon, Republic of Korea) (Figure 5b). The peri-implant defects were covered with a resorbable collagen membrane (Genoss, Suwon, Republic of Korea); however, the lateral window was not covered (Figure 5c) and the flap was primarily closed (Figure 5d). Postoperative medication was prescribed for 2 weeks, as in Case 1. In clinical findings of 6 months after the surgery, the surgical site was healed uneventfully (Figure 5e). During implant uncovering, the peri-implant gap defects were well regenerated with hard tissues and an additional Ø 4.3 × 10 mm implant was placed at the maxillary right second premolar site. The healing abutments were connected (Figure 5f), and the flap was closed (Figure 5g). The prosthesis was delivered 2 months later (Figure 5h,i).

**Figure 6:**
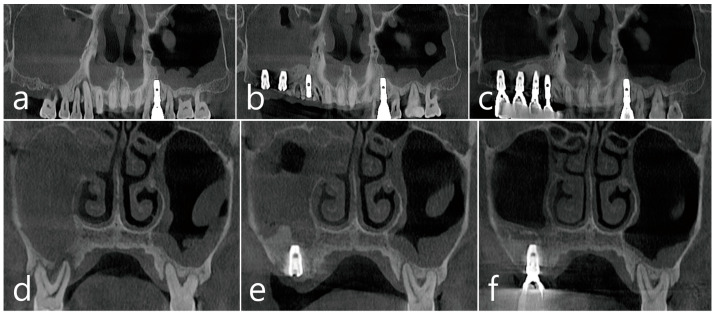
Case 2. Panoramic CBCT images before surgery (**a**), immediately after surgery (**b**), and 1 year after the prosthetic loading (**c**). Coronal CBCT images before surgery (**d**), immediately after surgery (**e**), and 1 year after prosthetic loading (**f**). Sinus opacification and nasal polyp disappeared in the images obtained 1 year after the loading. CBCT; cone beam computed tomography.

Panoramic CBCT images were obtained before the surgery (Figure 6a), immediately after implant placement with the MSFA (Figure 6b), and 1 year after prosthetic loading (Figure 6c). While the opacified sinus was visible before and immediately after the surgery, resolution of the lesion and maintenance of the augmented sinus floor surrounding the implant were noted in the radiographic view after 1 year. Coronal CBCT images obtained before (Figure 6d) and immediately after the surgery (Figure 6e) also showed sinus opacification and nasal polyps, which disappeared in the view obtained 1 year after loading (Figure 6f).

## 3. Discussion

Rehabilitation of edentulous ridges with dental implants is a common treatment strategy. Owing to the rapid advancements in surgical techniques, devices, and materials, MSFA has been widely used to address severely reduced ridge height in posterior maxilla and to facilitate implant placement. In addition, development in the implant surface texture with a roughened surface, representatively the sandblasting-and-acid-etching (SLA) surface, has shown enhanced osseointegration at earlier time points and adequate stability in grafted sinus or poor bone quality sites with which the posterior maxilla is often involved [24,25]. Dental clinicians frequently encounter and manage sinus pathologies, such as severe mucosal thickening and maxillary sinusitis, during MSFA planning. Although reports indicate successful outcomes of MSFA in cases of sinus membrane thickening and minor sinus pathologies [8,22,23,26], the patient in the present case report had preexisting CMS, observed as unilateral maxillary sinus opacification throughout the cavity and blockage of the natural ostium on wide-range CBCT images. Symptoms including congestion and nasal discharge were accompanied by pathological radiographic appearance, necessitating consultation with an otolaryngologist for treatment prior to MSFA or implant placement. Despite antibiotic therapy, soft tissue lesions showed minimal resolution, and FESS was recommended for these patients. FESS is the current gold standard for restoring clearance and ventilation of the maxillary sinus and resolving sinonasal conditions [8]. It is also indicated for patients with drug resistance, as well as those with nasal polyposis and benign or malignant tumors. However, in case of odontogenic CMS due to treatable dental pathology such as tooth extraction, there is very little evidence to support whether dental treatment or FESS should be performed primarily [12]. FESS is also involved with hospitalization, general anesthesia, economic burdens and longer treatment periods compared to the management with surgical drainage during MSFA at the dental clinic.

CMS is known to be the incompletely resolved acute sinusitis that prolongs the course of infection [8,11]. Case 1 was considered as the rhinogenous origin, as the patient showed a full edentulous ridge in maxilla that excluded dental pathology. Suppurative sinusitis is involved with mucosal factors such as nasal polyposis, which results in the stenosis of the osteomeatal complex due to the swelling of the sinus mucosa [27], which is in correspondence with Case 1. Apart from the local host factors, such as anatomic structures and acquired mucociliary dysfunction, systemic host factors and environmental factors, including microorganisms, are also known to be associated with CRS, which make etiologies of the disease heterogeneous in nature [28]. The microbiology of CRS demonstrates a wide range of bacteria predominantly of *Staphylococcus aureus*, coagulase-negative *Staphylococcus*, and various gram-negative rods [29]. The role of microbial infection and biofilms in the pathophysiology of CRS is unclear, but they may contribute to the propagation of disease. Empiric antimicrobial therapy is often used to target against the broad spectrum of bacteria and the first-line antibiotics include amoxicillin-clavulanate which was used in the present case report with short-term periods [28]. However, long-term empiric application has concern for the emergence of antibiotic resistance especially in the recalcitrant patient and microbial culture can provide guide for accurate treatment [30].

On the other hand, CMS of dental origin was suspected in Case 2 due to an advanced apical lesion adjacent to the sinus floor in the maxillary posterior teeth. Removal of dental pathology, such as extraction, endodontic treatment, or repair of oroantral fistulas, should be included in the management of odontogenic CMS. Reports indicate that 70–77% of odontogenic maxillary sinusitis cases resolve after 3 months with the extraction of the causative tooth [31,32]. It is also described that dental treatment is enough for full recovery of CMS when the pathology is limited to the maxillary sinus, but the persistence of malodor 2 weeks after eliminating odontogenic problems strongly indicate the need for surgical sinus intervention [31]. Park et al. reported that in terms of sinus mucosal thickening of odontogenic origin, surgical drainage has a greater reduction effect than the extraction of the causative tooth [9]. Waiting for the reduction of mucosal thickening after tooth extraction inevitably prolongs the treatment period, and a failure rate of close to 30% becomes a burden for both the operator and patient. Therefore, we decided to extract the causative tooth and perform surgical drainage simultaneously in Case 2.

Mucociliary clearance (MCC) and patency of the ostium play important roles in host defense mechanisms that maintain homeostasis in the maxillary sinus [33,34]. MCC allows ventilation and removal of the mucus and foreign bodies by the ciliary movement of the pseudostratified ciliated columnar epithelium lining the inner Schneiderian membrane, directing them through the ostium to the middle meatus of the nasal cavity [34,35]. Irregular mucosal appearance (>5 mm) and circumferential or complete mucosal thickening are associated with an increased risk of sinus obstruction [36]. It has been suggested that the height of sinus mucosal thickening exceeding a certain threshold of the entire height of the maxillary sinus could be a potential risk factor for ostial obstruction [8,23]. Based on the clinical practice guidelines for the treatment of CMS, saline irrigation is initially performed for CMS and antibiotics are administered for 3 weeks to prevent acute exacerbations [37]. In the present cases, the otolaryngologist recommended FESS after 2 weeks of antibiotic treatment due to unchanged sinus opacification, although the patients’ symptoms were alleviated. No universally accepted consensus on the criteria that constitute maximal medical therapy and the timing of surgery is currently available, and appropriate criteria for FESS should be further defined to reduce unwarranted surgical variation [37].

As an intraoral intervention, the conventional Caldwell–Luc operation is used for the complete removal of maxillary sinus lesions to replace them with new mucosa; however, it is associated with increased postoperative trauma and patient morbidity [38]. In contrast, the modified/mini-Caldwell–Luc operation without inferior meatal antrostomy removes polypoid tissue and inflamed sinus mucosa but preserves the intact sinus mucosa as much as possible, thereby being less invasive and reducing postoperative complications [39]. However, these surgical interventions are associated with the loss of sinus mucosa to some extent and require longer healing periods until re-entry for MSFA. Surgical drainage and saline irrigation without excisional removal of the sinus mucosa were performed in the present cases, which could be more conservative than the aforementioned interventions. A previous retrospective study evaluated radiographic changes in sinus mucosal thickness after tooth extraction and drainage during lateral sinus augmentation [9]. Patients who had sinus mucosal thickening potentially due to odontogenic origin, but no self-reported sinonasal symptoms were included in the study. Surgical drainage was performed using a 21-gauge aspiration needle at the center of the elevated Schneiderian membrane during the lateral approach of MSFA. The sinus mucosal thickness was shown to have been significantly reduced after the extraction of compromised teeth, but drainage contributed more to the reduction than extraction, indicating the effective alleviation of inflammation by drainage. Augmented bone and implants showed 100% survival on average at 6-year follow-up.

In addition, a retrospective study including a group without unrepaired perforation of the Schneiderian membrane reported a greater reduction in sinus mucosal thickness compared to that of the non-perforated group [40]. Therefore, sinus drainage and saline irrigation through the perforated site, whether accidental or intentional, may help remove cystic or inflammatory fluid and reduce the bacterial burden [41], resulting in a decrease in postoperative sinus membrane thickness and alleviation of symptoms in patients with CMS. Drainage itself cannot widen the natural ostium or directly influence nasal polyps. Since endoscopic images of the maxillary sinus cavity are not available, there is limited visibility to control drainage. Despite these limitations, a previous study reported re-establishment of ostial patency after drainage in five out of six patients who had ostial obstruction preoperatively due to its effect on reducing sinus membrane thickness [9]. As the patients in the present case report had advanced sinus mucosal thickening, improved intraoral accessibility of instruments in various directions with sufficient depth into the sinus cavity was necessary to drain purulent exudate thoroughly and wash it out completely before simultaneous MSFA. Still, sinus drainage with spontaneous MSFA may not be feasible for routine application without proper experience due to the potential risks of graft displacement through perforated site, resultant contamination of graft material, and persisting postoperative sinusitis.

An intentional incision rather than a small puncture with the aspiration needle on the Schneiderian membrane was used in the present case to gain access for thorough drainage and saline irrigation. A large bony window was prepared, and a long incision was made in the upper portion of the exposed Schneiderian membrane using a surgical blade. Direct insertion and free access of the suction tip into the sinus cavity make it possible to control the contaminated area more easily. The one-bony-window technique, comprising a large window opening and intentional incisional perforation, was previously introduced to remove a large antral pseudocyst without rupture simultaneously with MSFA and showed favorable outcomes in implant survival with maintenance of augmented bone height [42]. Both patients in the present study showed a significant reduction in sinus mucosal thickness, confined to the area around the augmented bone, without clinical symptoms related to sinusitis after 1-year of surgery. In Case 1, the patient had a nasal polyp at the middle meatus that blocked the natural ostium preoperatively, and a decrease in the expanded polyp was observed after sinus drainage, suggesting an indirect positive effect on the nasal cavity through patency of the ostium.

Making an intentionally long-incised perforation in the Schneiderian membrane can be counterintuitive to clinicians, especially when simultaneous MSFA is planned. Some studies have demonstrated that sinus membrane perforation may lead to graft displacement, postoperative maxillary sinusitis, and compromised results in bone grafting and implant survival [43,44], whereas others have reported controversial opinions that perforation is not associated with postoperative infection or implant failure owing to advanced repair techniques [7,45]. In the present cases, careful detachment and elevation of the Schneiderian membrane at the sinus floor, distant from the perforated area, were performed after drainage. By relieving the tension on the sinus membrane, the perforation was reduced in size and positioned buccally with mucosal folding, without the additional use of a collagen barrier membrane. A Prichard elevator was inserted to cover the remaining perforated area during bone grafting to prevent the displacement of the particles into the sinus cavity. Immediate postoperative radiograph showed no notable leakage of graft materials, and maintenance of augmented bone height was observed after 1 year of loading. Although the patients in this study were treated with CMS and surgical drainage, the clinical outcomes were consistent with that of a previous study reporting that nonrepaired perforation of the Schneiderian membrane did not adversely affect the outcomes of MSFA [40].

This case report has limitations. The approach in the management of CMS with surgical drainage simultaneously with MSFA is the first of its kind, and supporting evidence is still lacking, as the fundamental articles related to this case report are mostly case reports and case series. Moreover, the number of cases was small, and the follow-up period was short. Case selection should be carefully considered for CMS in patients whose symptoms are mild, and infection is limited to the maxillary sinus. There is limited evidence to determine the critical extent or size of mucosal thickening that allows MSFA and implant placement. It is important to evaluate the height of soft tissue lesions relative to the entire height of the maxillary sinus to avoid blockage of the ostium after elevation of the Schneiderian membrane. Potent complications and recurrence of CMS regarding MSFA spontaneously with sinus drainage in long-term results should be evaluated.

## 4. Conclusions

Within the limitations of the present case report, thorough sinus drainage and saline irrigation during the lateral window approach enabled the resolution of sinus infection and prevented postoperative sinusitis in simultaneous MSFA with implant placement in patients with CMS. Favorable clinical outcomes of implant survival with maintenance of augmented sinus floor were observed during short-term observation periods.

## Data Availability

Data are contained within the article.
